# Supplementary material for: Effectiveness of artificial urinary sphincter to treat stress incontinence after prostatectomy: A meta-analysis and systematic review
Source: PLoS One. 2023 Sep 1;18(9):e0290949. doi: 10.1371/journal.pone.0290949 (PMC10473540; doi:10.1371/journal.pone.0290949)
Supplement: S1 Checklist — (DOCX) [file pone.0290949.s001.docx]

| **Section and Topic** | **Item #** | **Checklist item** | **Location where item is reported** |
| --- | --- | --- | --- |
| **TITLE** | | |  |
| Title | 1 | The report is identified as a meta-analysis and systematic review | 1 |
| **ABSTRACT** | | |  |
| Abstract | 2 | The structured abstract includes Background,Methods,Results,Conclusion | 1 |
| **INTRODUCTION** | | |  |
| Rationale | 3 | Described in the introduction | 2 |
| Objectives | 4 | Stated in the introduction | 2 |
| **METHODS** | | |  |
| Eligibility criteria | 5 | The specified research characteristics ( PICOS ) and the characteristics of the report ( such as retrieval time, language and publication status ) are used as the criteria for inclusion studies, and explanations are given. | 3 |
| Information sources | 6 | Describe the source of all literature information for each search and the final search results. | 4-5 |
| Search strategy | 7 | Search strategies were developed according to PICOS criteria ( population, intervention, control, outcome, and study design ). | 3 |
| Selection process | 8 | The two researchers identified and selected data from the study in detail and independently, and the differences were resolved by consensus or cooperation with the third member of the research team. | 3 |
| Data collection process | 9 | Two researchers extracted data from the study in detail and independently, and differences were resolved by consensus or in cooperation with a third member of the research team. | 3 |
| Data items | 10a | The outcome indicators that need to be collected are described in detail, and the definition of the outcome indicators is explained, including the author, the year of publication, age, follow-up time,Primary: complete dry rate (0 pads/day); Social dry rate(0~1 pad/day)；Secondary: differential pad count (after adjustment with respects to baseline), Quality of life. | 3 |
|  | 10b | Population:Male patients with mild, moderate or severe stress urinary incontinence after prostatectomy;Intervention:artificial urinary sphincter AMS 800™ (Boston Scientific, Boston, USA)。 | 3 |
| Study risk of bias assessment | 11 | Evaluate whether there is publication bias in the results of the study through the funnel plot independently. The symmetrical distribution of each index in the funnel plot indicates that there is no publication bias. In the Egger test, the results of the outcome indicators were quantitatively tested. When P < 0.05, it was suggested that there may be a greater possibility of publication bias. | 4 |
| Effect measures | 12 | The data of dry rate and social dry rate were converted into standard errors, and 95 % confidence intervals ( CIs : lower and upper limits ) were used for statistical evaluation. For daily use of pads and quality of life, the average value with standard deviation was calculated. | 4 |
| Synthesis methods | 13a | Describe the processes used to decide which studies were eligible for each synthesis (e.g. tabulating the study intervention characteristics and comparing against the planned groups for each synthesis (item #5)). | 4-5 |
|  | 13b | If the study did not report the mean and standard deviation, the mean and standard deviation were estimated from the sample size, median, range, or interquartile range. If a study reports the median and interquartile range ( IQR ), we assume that the median of the outcome variable is equal to the mean effect, and the width of the interquartile range is about 1.35 standard deviations. | 4 |
|  | 13c | Using Excel for data statistics | 4 |
|  | 13d | Meta-analysis was performed using Stata 17.0 software. Heterogeneity test : if I^2^<50 %, indicating high heterogeneity, using random effects model ; if I^2^ > 75 %, which indicates a high degree of heterogeneity, a random effects model is used. | 4 |
|  | 13e | Subgroup analysis was performed according to the severity of urinary incontinence. | 11-12 |
|  | 13f | The funnel plot was used to detect whether there was publication bias in the study results. | 4 |
| Reporting bias assessment | 14 | Sensitivity analysis of the main outcomes was performed using the article-by-article exclusion method. | 4 |
| Certainty assessment | 15 | Revised 18-item Delphi checklist was used to assess the quality of included studies. | 4 |
| **RESULTS** | | |  |
| Study selection | 16a | Presented in a flow chart : retrieval and screening process, the number of retrieved records to the final number of included studies. | 5-6 |
|  | 16b | Explain studies that met the inclusion criteria but were excluded. | 5-6 |
| Study characteristics | 17 | Table 3 shows the characteristics of each document extracted. | 7-9 |
| Risk of bias in studies | 18 | Publication bias test was performed on dry rate, social dry rate by making funnel plots and Egger test (Fig 6, Fig 7). Sensitivity analysis was performed on dry rate, social dry rate (Fig 8, Fig 9) using the one-by-one exclusion method. | 14-16 |
| Results of individual studies | 19 | Meta-analysis was performed on outcome indicators : dry rate ( Fig2 ), social dry rate ( Fig3 ), daily pad use ( Fig4 ), and quality of life ( Fig5 ). | 10-14 |
| Results of syntheses | 20a | The distribution of each index in the dry rate funnel plot was asymmetric, and the Egger test P = 0.04 < 0.05, suggesting that there may be bias (Fig 6). The symmetry of the funnel plot in the social dry rate was good, and the Egger test P = 0.376 > 0.05, suggesting that the possibility of publication bias was low. ( Fig 7 ). | 15 |
|  | 20b | Meta-analysis was performed using a random effect model. The drying rate was 52 % ( 95 % CI = 0.39-0.66 ). The social dry rate was 81 % ( 95 % CI = 0.73-0.89 ). The mean difference of comprehensive SMD was ( 2.68,95 % CI = 2.07-3.29 ). Quality of life, ICIQ-SF score group, comprehensive SMD mean difference was ( 1.77,95 % CI = 1.57 ~ 1.96 ) ; in the VAS score group, the mean difference of comprehensive SMD was ( 3.45,95 % CI = 2.92-3.99 ). The above ( P < 0.001 ) differences were statistically significant. | 10-14 |
|  | 20c | Subgroup analysis of dry rate and social dry rate was performed according to the severity of urinary incontinence. | 11,13 |
|  | 20d | The sensitivity analysis of dry rate,social dry rate ( Fig 8,Fig 9 ) was performed using the one-by-one exclusion method. | 16 |
| Reporting biases | 21 | Revised 18-item Delphi checklist ( S1Table ) was used to assess the quality of included studies. | 10 |
| Certainty of evidence | 22 | Present the results of outcome confidence evaluation | 10-13 |
| **DISCUSSION** | | |  |
| Discussion | 23a | Provide a general interpretation of the results in the context of other evidence. | 17-18 |
|  | 23b | Limitations of included studies. | 19 |
|  | 23c | Limitations of research analysis | 19 |
|  | 23d | The analysis of the summary of the results is given, and the suggestions for future research are put forward. | 19 |
| **OTHER INFORMATION** | | |  |
| Registration and protocol | 24a | Registration does not apply |  |
|  | 24b | Not prepared a protocol |  |
|  | 24c | Not prepared a protocol |  |
| Support | 25 | There is no source of economic support |  |
| Competing interests | 26 | No conflict of interest between authors | 19 |
| Availability of data, code and other materials | 27 | Delphi checklist | 10 |

*From:*  Page MJ, McKenzie JE, Bossuyt PM, Boutron I, Hoffmann TC, Mulrow CD, et al. The PRISMA 2020 statement: an updated guideline for reporting systematic reviews. BMJ 2021;372:n71. doi: 10.1136/bmj.n71

For more information, visit: <http://www.prisma-statement.org/>
