# Supplementary material for: Effectiveness of artificial urinary sphincter to treat stress incontinence after prostatectomy: A meta-analysis and systematic review
Source: PLoS One. 2023 Sep 1;18(9):e0290949. doi: 10.1371/journal.pone.0290949 (PMC10473540; doi:10.1371/journal.pone.0290949)
Supplement: S1 Table — (DOCX) [file pone.0290949.s002.docx]

**S 1 The summarized results of quality assessment for insuifluded studies.**

| 1at Author (year) | 1 | 2 | 3 | 4 | 5 | 6 | 7 | 8 | 9 | 10 | 11 | 12 | 13 | 14 | 15 | 16 | 17 | 18 |
| --- | --- | --- | --- | --- | --- | --- | --- | --- | --- | --- | --- | --- | --- | --- | --- | --- | --- | --- |
|  | Is the hypothesis/aim/objective of the study clearly stated in the abstract, introduction, or methods section? | Are the characteristics of the participants included in the study described? | Were the cases collected in more than one centre? | Are the eligibility criteria (inclusion and exclusion criteria) to entry the study explicit and appropriate? | Were participants recruited consecutively? | Did participants enter the study at a similar point in the disease? | Was the intervention clearly described in the study? | Were additional interventions clearly reported in the study? | Are the outcome measures clearly defined in the introduction or methods section? | Were relevant outcomes appropriately measured with objective and/or subjective methods? | Were outcomes measured before and after intervention? | Were the statistical tests used to assess the relevant outcomes appropriate? | Was the length of follow-up reported? | Was the lost to follow-up reported? | Does the study provide estimates of the random variability in the data analysis of relevant outcomes? | Are adverse events reported? | Are the conclusions of the study supported by results? | Are both Conflicts of Interest and source of support for the study reported? |
| Sacco（2021） | Y | Y | N | Y | Y | Y | Y | N | Y | Y | Y | Y | Y | Y | Y | Y | Y | Y |
| Kuznetsov（2000） | Y | N | N | Y | Y | Y | Y | N | Y | Y | N | Y | N | Y | Y | Y | Y | N |
| Imamoglu（2005） | Y | N | N | Y | N | Y | Y | N | Y | Y | Y | Y | Y | N | Y | N | Y | N |
| Ahyai（2016） | Y | Y | N | Y | N | Y | Y | N | Y | Y | Y | Y | Y | Y | Y | Y | Y | Y |
| Sotelo（2008） | Y | N | N | Y | N | Y | Y | N | Y | Y | Y | Y | Y | N | Y | Y | Y | Y |
| Serra（2016） | Y | Y | N | Y | Y | Y | Y | Y | Y | Y | Y | Y | Y | N | Y | N | Y | Y |
| Grabbert（2019） | Y | Y | Y | Y | N | Y | Y | N | N | Y | Y | Y | Y | Y | Y | N | Y | Y |
| Fan Zhang（2022） | Y | Y | N | Y | N | Y | Y | N | Y | Y | Y | Y | Y | N | Y | Y | Y | Y |
| Trigo（2008） | Y | Y | N | Y | Y | Y | Y | N | Y | Y | Y | Y | Y | N | Y | Y | Y | N |
| Fan Zhang（2018） | Y | Y | N | Y | N | Y | Y | N | Y | Y | Y | Y | Y | N | Y | N | Y | N |
| Lin-Feng Meng（2019） | Y | N | N | Y | N | Y | Y | N | Y | Y | Y | Y | Y | N | Y | N | Y | Y |
| Fan Zhang（2022） | Y | Y | N | Y | N | Y | Y | N | Y | Y | Y | Y | Y | N | Y | Y | Y | Y |
| Maurer（2019） | Y | Y | Y | Y | N | Y | Y | Y | Y | Y | N | Y | Y | N | Y | Y | Y | Y |
| Maure（2020） | Y | Y | N | Y | N | Y | Y | Y | Y | Y | N | Y | Y | Y | Y | Y | Y | Y |
| O'Connor (2008) | y | N | N | Y | N | Y | Y | N | Y | Y | Y | Y | Y | N | Y | Y | Y | N |
| Constable (2022) | Y | N | N | Y | N | Y | Y | N | Y | Y | Y | Y | Y | N | Y | Y | Y | N |
| Fan Zhang（2016） | Y | N | N | Y | N | Y | Y | N | Y | Y | Y | Y | Y | N | Y | Y | Y | N |
| Mottet (1998) | Y | N | Y | Y | N | Y | Y | N | Y | Y | N | Y | N | N | Y | Y | Y | N |
| O'Connor (2007) | Y | Y | N | Y | N | Y | Y | Y | Y | Y | Y | Y | Y | Y | Y | Y | Y | N |

Y: yes; N: No; NA: not available.
