# Supplementary material for: Effectiveness of artificial urinary sphincter to treat stress incontinence after prostatectomy: A meta-analysis and systematic review
Source: PLoS One. 2023 Sep 1;18(9):e0290949. doi: 10.1371/journal.pone.0290949 (PMC10473540; doi:10.1371/journal.pone.0290949)
Supplement: S2 Table — (DOCX) [file pone.0290949.s003.docx]

**S 2 Search strategy**

| Database | Search Details | Results |
| --- | --- | --- |
| PubMed | (("urinary sphincter, artificial"[MeSH Terms] OR "artificial urinary sphincter"[Title/Abstract] OR (("Sphincter"[All Fields] OR "sphincter s"[All Fields] OR "sphincteral"[All Fields] OR "sphincteric"[All Fields] OR "Sphincters"[All Fields]) AND "artificial urinary"[Title/Abstract]) OR (("urinary tract"[MeSH Terms] OR ("Urinary"[All Fields] AND "tract"[All Fields]) OR "urinary tract"[All Fields] OR "Urinary"[All Fields]) AND "sphincters artificial"[Title/Abstract])) AND "urinary incontinence"[MeSH Terms] AND "male"[MeSH Terms]) AND (clinicaltrial[Filter] OR randomizedcontrolledtrial[Filter]) | 30 |
| web of science | (((((TS=(urinary sphincter, artificial)) OR TS=(Artificial Urinary Sphincter)) OR TS=(Sphincter, Artificial Urinary)) OR TS=(Urinary Sphincters, Artificial)) AND TS=(urinary incontinence)) AND TS=(male) and 论文 (文献类型) | 387 |
| Embase | #10. #9 AND ('controlled clinical trial'/de OR 'randomized controlled trial'/de)  #9. #7 AND #8  #8. 'male'  #7. #5 AND #6  #6. urinary AND incontinence  #5. #1 OR #2 OR #3 OR #4  #4. urinary AND sphincters, AND artificial  #3. sphincter, AND artificial AND urinary  #2. artificial AND urinary AND sphincter  #1. urinary AND sphincter, AND artificial | 38 |
| cochrane | #1 MeSH descriptor: [Urinary Sphincter, Artificial] explode all trees  #2 Artificial Urinary Sphincter 83  #3 Sphincter, Artificial Urinary 83  #4 Urinary Sphincters, Artificial 17  #5 #1 OR #2 OR #3 83  #6 urinary incontinence 8269  #7 male 803987  #8 #5 AND #6 AND #7 48 | 48 |
| CNKI,WanFang Data, VIP databases | (主题=人工尿道括约肌 ) OR (篇关摘=尿道括约肌, 人工) AND (主题=压力性尿失禁) | CNKI:17  WanFang Data:12  VIP databases:7 |
